# The developmental transcriptomes of two sea biscuit species with differing larval types

**DOI:** 10.1186/s12864-018-4768-9

**Published:** 2018-05-18

**Authors:** Anne Frances Armstrong, Richard K. Grosberg

**Affiliations:** 1 Center for Population Biology, University of California, Davis, 1 Shields Avenue, Davis, CA 95616 USA; 2 California Academy of Sciences, 55 Music Concourse Drive, San Francisco, CA 94118 USA; 3 Coastal and Marine Sciences Institute, University of California, Davis, 1 Shields Avenue, Davis, CA 95616 USA

**Keywords:** Transcriptomics, RNAseq, Larval development, Echinoderm, Life-history, Planktotrophy, Facultative feeding

## Abstract

**Background:**

Larval developmental patterns are extremely varied both between and within phyla, however the genetic mechanisms leading to this diversification are poorly understood. We assembled and compared the developmental transcriptomes for two sea biscuit species (Echinodermata: Echinoidea) with differing patterns of larval development, to provide a resource for investigating the evolution of alternate life cycles. One species (*Clypeaster subdepressus*) develops via an obligately feeding larva which metamorphoses 3–4 weeks after fertilization; the other (*Clypeaster rosaceus*) develops via a rare, intermediate larval type—facultative feeding— and can develop through metamorphosis entirely based on egg provisioning in under one week.

**Results:**

Overall, the two transcriptomes are highly similar, containing largely orthologous contigs with similar functional annotation. However, we found distinct differences in gene expression patterns between the two species. Larvae from *C. rosaceus*, the facultative planktotroph, turned genes on at earlier stages and had less differentiation in gene expression between larval stages, whereas, *C. subdepressus* showed a higher degree of stage-specific gene expression.

**Conclusion:**

This study is the first genetic analysis of a species with facultatively feeding larvae. Our results are consistent with known developmental differences between the larval types and raise the question of whether earlier onset of developmental genes is a key step in the evolution of a reduced larval period. By publishing a transcriptome for this rare, intermediate, larval type, this study adds developmental breadth to the current genetic resources, which will provide a valuable tool for future research on echinoderm development as well as studies on the evolution of development in general.

**Electronic supplementary material:**

The online version of this article (10.1186/s12864-018-4768-9) contains supplementary material, which is available to authorized users.

## Background

Over the last century, echinoid echinoderms have come to represent an iconic model system for studying questions about the evolution of development, due in large part to their diverse larval forms, even among closely related species [1–13]. However, only recently has it become possible to decipher the genetic changes underlying evolutionary shifts in developmental mode [14–18]. Here, we present the developmental transcriptomes of two closely related echinoids as a foundation for analyzing how patterns of gene expression change during the course of larval evolution.

Despite their great morphological diversity, marine larvae are typically classified into two groups: planktotrophic larvae, that develop from small eggs and must feed in order to develop through metamorphosis, and lecithotrophic larvae, that do not feed as larvae but instead develop entirely based on energy provided in their large yolky eggs [7]. Feeding, planktotrophic larvae presumably represent the ancestral state for all echinoderms, whereas non-feeding larvae have evolved convergently dozens of times [13, 19]. While the evolution of non-feeding larvae from feeding larvae involves many morphological and functional changes, including (1) an increase in egg size; (2) reduction in developmental time; (3) accelerated development of a juvenile body; and (4) the reduction—or even complete loss—of larval feeding structures, the transition can occur rapidly [20–27].

There are a handful of intermediate larval types between these two extreme modes, which have proven invaluable for understanding the speed, manner, and order in which developmental changes occur during the evolution of non-feeding forms [26, 28–30]. The sea biscuit *Clypeaster rosaceus* is perhaps the best-studied echinoderm with an intermediate larval type [27, 31–33]. Like obligate planktotrophs, including its closely related congener *C. subdepressus*, *C. rosaceus* larvae develop through a pluteus larval form with functioning feeding structures [31]. However, unlike obligately planktotrophic larvae, *C. rosaceus* larvae develop from large eggs, can complete metamorphosis without feeding [33] and develop in under a week, as opposed to the three weeks *C. subdepressus* takes [31, 34]. Furthermore, *C. rosaceus* larvae begin forming structures that develop into a juvenile shortly after gastrulation, like species with non-feeding development, but unlike species with obligately feeding larvae that typically do not produce juvenile structures until late larval stages [26]. Lastly, the larval feeding structures of *C. rosaceus* are smaller and less efficient relative to obligate planktotrophs [31, 35]. Consequently, studies on the development of *C. rosaceus*, as well as other species with intermediate larval types, fill a void in our understanding of the order and timing of morphological and genetic changes during the evolutionary transition from feeding to non-feeding larvae [28, 29].

Published transcriptomes for echinoderm species with both planktotrophic [16, 17, 36, 37] and lecithotrophic [38] development reveal dramatic changes in gene expression and gene regulatory networks between the two [17]. However, there are currently no assembled transcriptomes for species with intermediate larval types. We therefore assembled transcriptomes for *C. rosaceus*, along with a closely related congener, *C. subdepressus* [33]. These two species are an ideal study system for understanding the order and manner in which divergent patterns of gene expression accumulate during the evolutionary transition between larval types not only because of their close phylogenetic relationship, but also due to the extensive previous work on their early development [31, 32, 35], and the fact that they can produce viable hybrid larvae [34].

## Methods

### Tissue collection

We collected adult sea biscuits (*C. rosaceus* and *C. subdepressus*) from subtidal sand beds at Bocas Del Toro, Panama during the months of August and September 2013, and maintained them in ambient conditions in outdoor flow-through tanks at the Bocas Del Toro Research Station of the Smithsonian Tropical Research Institute. To generate embryos and larvae, we established three conspecific replicate crosses for each species. In each replicate cross, we fertilized eggs from a single female with sperm from a single male in 100 ml finger bowls, so that the larvae of each species were full siblings within a replicate, but were unrelated across replicates.

We reared larvae according to the methods described in Armstrong and Lessios [34]. We set up 1000 ml tri-pour beakers with sea-water filtered through a 0.45μm membrane. After embryos hatched, we added 200 larvae of either *C. rosaceus* or *C. subdepressus* to each beaker. We maintained larvae on a Strathmann stirring rack [8] at ambient temperature conditions. Every other day, we changed 80% of the water in each beaker and fed larvae a mix of the two algal species *Isochrysis galbana* and *Rhodomonas* spp. at a concentration of 5000 cells/ml.

We collected RNA samples at four developmental stages: unfertilized eggs (0 h post-fertilization: 0 hpf), gastrulae (20 hpf for both species), four-armed larvae (2 days post-fertilization (dpf) in *C. rosaceus*; 3 dpf in *C. subdepressus*), and 8-armed larvae (4 dpf in *C. rosaceus*; 10 dpf in *C. subdepressus*) [developmental staging from Armstrong and Lessios [34]]. For each RNA sample, we preserved 100 individuals (eggs, gastrulae, or larvae) in RNAlater. We stored the samples at −20 °C while at Bocas Del Toro, followed by −80 °C for long-term storage at UC Davis.

### RNA extraction and sequencing

We extracted total RNA using Trizol, followed by a DNAse treatment using Ambion’s Turbo DNA-free kit. We quantified RNA using a Nanodrop and assessed RNA quality on an Agilent 2100 Bioanalyzer; we discarded any RNA samples with a RNA integrity number (RIN) < 7. From the resulting RNA samples, we created six replicate RNAseq libraries (two libraries for each of the three replicate experiments) for each developmental stage in each species, following standard protocols with the NEB Ultra Directional kit. We multiplexed the libraries together by using NEB multiplex primers and sequenced the libraries across three lanes of Paired-End100 on an Illumina HiSeq2500 machine at the UC Davis Genome Center.

### Assembly and annotation

We removed adapter sequences and low-quality reads from the raw Illumina reads with the program Trimmomatic (v0.36) using default parameters; we retained sequences that were at least 36 bp after trimming. We combined all of the remaining reads for each species and assembled transcriptomes using Trinity (v2.4.0) set to default parameters with a minimum contig length of 300 bp [39]. To account for redundant transcripts due to within-species polymorphisms or sequencing errors, we clustered the resultant Trinity outputs at 97% similarity using the program CD-hit (v4.6.5) [40]. For each clustered transcriptome, we identified contigs with coding potential through the program TransDecoder [41]. Finally, we used the script CompareContamSeq.pl from the Transcriptome Utilities package to remove contigs originating from bacterial and viral contaminants [42].

We used the programs CEGMA (Core Eukaryotic genes mapping approach) [43] and BUSCO (Benchmarking universal single-copy orthologs) [44] to assess the completeness of core eukaryotic and metazoan proteins, respectively, in each transcriptome. To identify orthologous contigs between the transcriptomes, we used the program OrthoFinder [45] with default parameters and compared our two assemblies along with the purple urchin transcriptome, *Strongylocentrotus purpuratus* (downloaded from *echinobase.org*) [46]. We functionally annotated the transcriptomes using the program Blast2GO (v4.1) [47] with the UniProt BLAST database. We ran the program Interpro scan to obtain functional information, including protein families, Panther biological process categories [48], and gene ontology (GO) terms [49]. We compared the overall GO distribution between the two transcriptomes using the R package GOstats to test for any significantly enriched biological process GO terms [50].

### Gene expression analysis

For differential expression analysis, we only retained libraries with > 3 million reads after quality filtering. We mapped our filtered reads back to their respective transcriptome using the program RSEM [41] to obtain expression tables. We normalized the data with a TMM (trimmed mean of m-values) method [51]. Using the normalized count data, we analyzed the Euclidean distances between the libraries from each species using a heatmap in DESeq [52]; we also set up a generalized linear model in EdgeR [53] with developmental stage and replicate experiment as fixed effects. From this model, we identified genes that were significantly differentially expressed in each species between developmental stages, with a false discovery rate of 0.05. Using the resulting lists of up-regulated genes at each stage, we identified any biological process GO terms that were over expressed between developmental time points. We used a hypergeometric test in the R package GOstats for this analysis [50].

## Results

### Assemblies

**Fig. 1:**
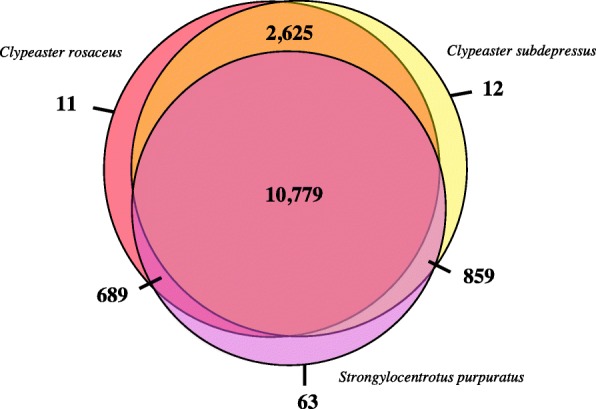
Summary of orthogroups between *Clypeaster rosaceus* (red), *C. subdepressus* (yellow), and *Strongylocentrotus purpuratus* (purple). Orthogroups with all three species present are shown overlapping in the middle (10,779). Orthogroups shared exclusively by the *Clypeaster* species are shown in orange (2625)

Using three lanes of HiSeq PE100, we generated 240 and 260 million reads for *C. rosaceus* and *C. subdepressus*, respectively, with a mean phred quality score > 36. After removing low-quality reads and adapters in trimmomatic, we retained > 95% (237 and 252 million reads, respectively) to assemble each transcriptome. The final assemblies had 33,190 and 36,059 contigs for *C. rosaceus* and *C. subdepressus*, with N50’s of 2769 bp and 2430 bp, respectively (Table 1). Both transcriptomes contained complete orthologs for between 96 and 99% of core eukaryotic and metazoan proteins when analyzed in the programs CEGMA and BUSCO respectively (Table 1). We identified orthologs between the two transcriptomes, along with the purple sea urchin transcriptome, using OrthoFinder (Emms and Kelly 2015). Orthofinder generated 15,038 orthogroups with 10,779 (72%) containing all three species (Fig. 1). The two *Clypeaster* species shared an additional 2625 orthogroups exclusively with each other, more than triple the number of orthogroups either species exclusively shared with *S. purpuratus* (Fig. 1).

**Table 1:** Assembly statistics for final transcriptomes of *C. rosaceus* and *C. subdepressus*

	Species	
	*Clypeaster rosaceus*	*Clypeaster subdepressus*
Number of Contigs	33,190	36,059
Total Nucleotides	66,040,159	60,049,487
N50 (bp)	2769	2430
GC Content	48.2%	49.2%
BUSCO *(Complete, Fragmented, Missing)*	97.2%, 1.9%, 0.9%	96.8%, 2.1%, 1.1%
CEGMA *(Complete, Fragmented, Missing)*	99.2%, 0%, 0.8%	99.2%, 0.4%, 0.4%

**Fig. 2:**
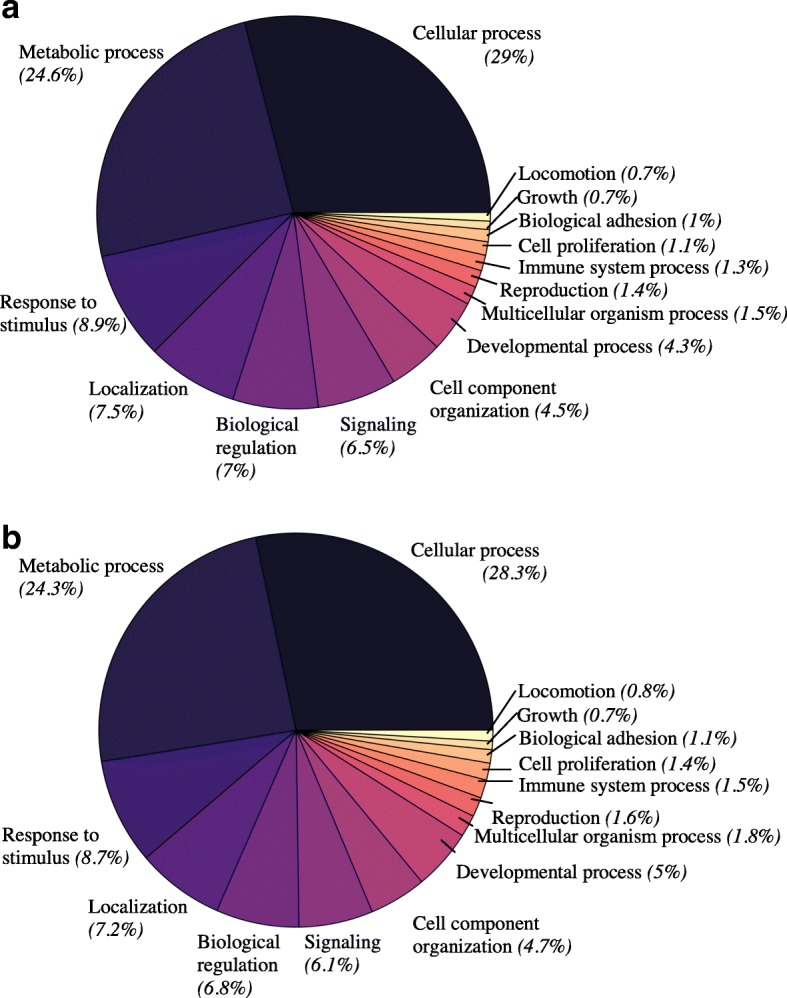
Distribution of biological process gene ontology terms in each transcriptome. The category and percent of GO terms belonging to that category are labeled for both *Clypeaster rosaceus* (**a**) and *C. subdepressus* (**b**)

Our BLAST search yielded at least one significant hit for 25,440 (77%) *C. rosaceus* contigs and 28,947 (80%) *C. subdepressus* contigs. Additionally, we obtained at least one GO term for 16,240 (49%) *C. rosaceus* contigs and 17,278 (48%) *C. subdepressus* contigs. Our analysis revealed no significant enrichment for any biological process GO terms between the two transcriptomes (Fig. 2). In both transcriptomes, cellular process, metabolic process, and response to stimulus were the largest GO terms, respectively (Fig. 2).

### Differential gene expression

After quality filtering and discarding any libraries with < 3 million reads, we retained 20 libraries per species, consisting of at least four replicates per developmental stage in each species. We used these libraries to analyze differential expression across developmental stages within each species.

**Fig. 3:**
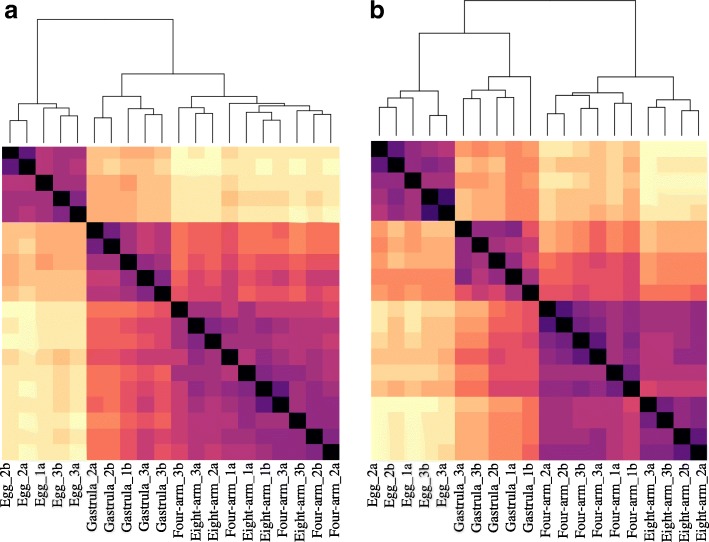
Heatmaps of the Euclidean distance between samples for each *Clypeaster rosaceus* (**a**)*,* and *C. subdepressus* (**b**). Dark purple colors represent highly similar samples, while lighter, yellow colors reflect more distant samples. The order of samples is denoted below each column with the sample names indicating first the developmental stage, followed by experiment (1–3), and replicate within experiment (a or b). The branching pattern at the top of each plot represents relative distances between samples

To determine which libraries were most similar, we analyzed the Euclidean distance between the samples from each species, using the heatmap function in DESeq [52]. From this analysis it was evident that, in both species, the eggs were the most distinct developmental time point from all other samples, followed by the gastrulae, and subsequently the two larval stages clustered together (Fig. 3). In *C. subdepressus*, the larval stages clearly clustered into two separate groups based on developmental stage. However, in the *C. rosaceus* samples, both larval stages were intermixed without a clear pattern. In both species, there was an effect of experiment, as replicates from the same experiment generally clustered together.

**Fig. 4:**
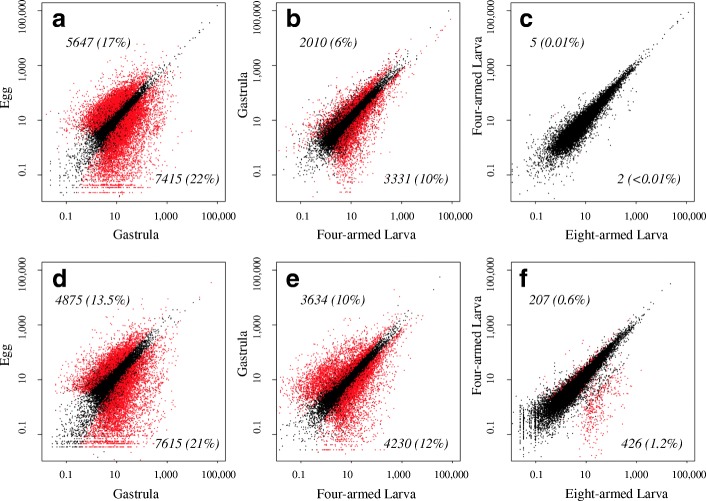
Scatterplots of stage specific gene expression. **a-c** *Clypeaster rosaceus*, (**d-f**) *C. subdepressus*. Expression levels are displayed in counts per million contrasted pairwise between developmental stages as labeled on the x- and y-axes. Red points represent genes identified as significantly differentially expressed, using a false discovery rate of 0.05, between stages while black dots are not significantly different. The number (and percent) of differentially expressed genes is indicated in each plot

Next, we identified differentially expressed genes (DEGs) using a generalized linear model in EdgeR, with both developmental stage and replicate experiment as fixed effects (Fig. 4). In both species, the greatest differences in expression occurred between the egg and gastrula stages, followed by the gastrula to 4-armed larva; and the smallest amount of differential expression occurred between the 4-arm and 8-arm larval stages (Fig. 4). More genes were differentially expressed during the egg-to-gastrula transition in *C. rosaceus* relative to *C. subdepressus*. In *C. rosaceus* 39.4% (13,062) of genes were expressed at statically different levels between the egg and gastrula stage as opposed to 34.6% (12,490) in *C. subdepressus*. However, the opposite was true in the other two contrasts (gastrula to 4-armed larva, and 4-armed to 8-armed larva) where there were more DEGs in *C. subdepressus* relative to *C. rosaceus*. This was most striking in the comparison between the four and eight-armed larvae, where only seven (0.02%) genes were differentially expressed in *C. rosaceus* whereas 633 genes (1.8%) were in *C. subdepressus*. There was a significant effect of replicate experiment, with 4.7% of *C. rosaceus* genes and 4.8% of *C. subdepressus* genes showing differences in gene expression between our three replicates.

We used the R-package GOstats to identify GO terms that were overrepresented in each list of differentially expresses genes at each developmental stage. This analysis revealed more significantly overrepresented GO terms at every developmental stage of *C. subdepressus* than in *C. rosaceus* (Additional file 1: Table S1). There was a high amount of overlap in the enriched GO categories between species at both the egg and gastrula stages; over 90% of significantly enriched GO terms in the eggs or gastrulae of *C. rosaceus* were also significantly enriched at the same stages of *C. subdepressus*. This trend, however, did not hold true when comparing significant GO terms in the larval stages (Additional file 1: Table S1).

In the eggs of both species, the GO terms with the greatest overrepresentation were related to protein modification, cell communication, signaling, and regulation of biological processes (GO:0036211, GO:0050794, GO:0051716, GO:0050789, GO:0007154, GO:0023052). In the gastrulae of both species the GO terms most enriched were related to translation, gene expression, biosynthetic processes (including ribosome biogenesis), mRNA processing, and protein folding (GO:0006412, GO:0010467, GO:0009058, GO:0022613, GO:0042254, GO:0006397). At the 4-arm larval stage, the only GO term enriched in both species was for transmembrane transport (GO:0055085). Additionally, cell communication, signaling, and cell cycle were enriched in *C. subdepressus* 4-armed larvae (GO:0007154, GO:0023052; GO:0007049, GO:0000278), whereas in *C. rosaceus* biosynthetic and metabolic processes were enriched (GO:0005975, GO:0044249, GO:0043043). At the 8-arm larval stage of *C. subdepressus,* metabolic processes were statistically enriched (GO:0044237, GO:0008152, GO:0006629), whereas no GO terms were significantly enriched in the 8-arm stage of *C. rosaceus* (for a full list of significant GO terms see Additional file 1: Table S1).

## Discussion

The developmental transcriptomes we assembled for *C. rosaceus* and *C. subdepressus* are highly consistent with one another in terms of assembly statistics, functional annotation and completeness of core eukaryotic proteins. The majority of contigs had orthologs in the other transcriptome, including many shared orthogroups that were absent from the *Strongylocentrotus purpuratus* outgroup. The two *Clypeaster* species diverged about 8 million years ago [33], which is consistent with the low level of differentiation between their transcriptomes.

Both transcriptomes showed the largest differences between ontological states in gene expression at the earliest developmental stages, with diminishing numbers of DEGs as development progressed. This result is expected, as a vast amount of developmental and morphological rewiring occurs between the egg and gastrula stage, compared to the minimal differences between larval stages [38, 54]. In echinoids, zygotic transcription begins as early as the first cleavage [55–57], so that while the transcripts in unfertilized eggs are entirely maternally deposited, the transcripts in the gastrulae should be entirely zygotic. Additionally, many studies report a spike of transcripts involved in ribosome production, translation, and protein synthesis in early embryonic phases, as we observed in the gastrulae of both species [36, 57]. Overall, these patterns were consistent between our species of *Clypeaster*, and with studies of embryonic development in other species [18, 36, 37].

Obligately planktotrophic larvae, which must acquire food to develop, focus their energy on feeding and producing feeding structures during the four-arm larval stage, while postponing the production of a juvenile rudiment [26, 58]. Alternatively, the facultatively feeding larvae of *C. rosaceus* begin developing juvenile structures shortly after gastrulation rather than solely devoting their energy towards feeding at early larval stages [26]. Due to these developmental differences, gene expression should be more dynamic in *C. subdepressus* where the embryonic and larval stages have more distinct functions. Not surprisingly, the most striking difference we found between these two species was that *Clypeaster subdepressus* displayed more dynamic, stage-specific, gene expression patterns—as indicated by our differential expression results, cluster analysis, and GO analysis—than *C. rosaceus*, particularly during the later larval stages. The only time point where we observed a higher turnover of differentially expressed genes in *C. rosaceus* relative to *C. subdepressus* was between the egg and gastrula stages; consistent with the fact that facultatively feeding larvae initiate the development of a juvenile body at the gastrula stage, whereas obligately planktotrophic larvae do not begin this process until late larval stages. Thus, just as accelerated developmental processes are one of the first steps in the evolution of non-feeding larvae [26, 27], our data suggest that accelerated gene activation is a key initial step in the loss of larval feeding.

## Conclusions

The transcriptomes we present add both developmental and phylogenetic breadth to the currently available larval transcriptomes. Both assemblies were highly complete and similar to one another but displayed distinct expression patterns that fit with our understanding of the developmental differences between these two species. Our results indicate that similar genes are expressed throughout development in *C. rosaceus* and *C. subdepressus*. However, the timing of gene expression seems accelerated in the intermediate, facultatively feeding larvae, of *C. rosaceus* relative to the obligately feeding, *C. subdepressus* larvae. Future studies using additional species with intermediate larval types, such as the heart urchin *Brisaster latifrons* [11], could further explore whether these differences are indeed related to life history rather than species-specific differences. However, as *C. rosaceus* and *B. latifrons* are the only with only two documented echinoids with facultatively feeding larval types, such a comparative analysis would be limited in scope. Alternatively, it is possible to hybridize *C. rosaceus* and *C. subdepressus*. Each reciprocal hybrid cross has a similar zygotic genome, with half of their DNA coming from each species, regardless of which species the egg or sperm came from. However, offspring developmental mode depends on which species provides the egg [34]. Thus, the hybrid offspring provide further contrasts of developmental type while accounting for species-specific genomic differences. As such, future studies using these hybrid offspring provide a unique opportunity to reveal critical insights into the patterns and regulatory mechanisms involved in larval evolution.

## Additional file

Additional file 1: **Table S1.** Text table listing every gene ontology term identified as significantly over expressed for each species at each developmental stage along with their *p*-values. (PDF 52 kb)

## Abbreviations

BLAST: Basic local alignment search tool
bp: base pair
BUSCO: Benchmarking universal single-copy orthologs
CEGMA: Core Eukaryotic genes mapping approach
DEG: Differentially expressed gene
DNA: Deoxynucleic acid
dpf: days post-fertilization
GO: Gene ontology
hpf: hours post-fertilization
RIN: RNA integrity number
RNA: Ribonucleic acid
TMM: Trimmed mean of m-values
