# Supplementary material for: The developmental transcriptomes of two sea biscuit species with differing larval types
Source: BMC Genomics. 2018 May 18;19:368. doi: 10.1186/s12864-018-4768-9 (PMC5960215; doi:10.1186/s12864-018-4768-9)
Supplement: Supplementary file 1 — Table S1. Text table listing every gene ontology term identified as significantly over expressed for each species at each developmental stage along with their p-values. (PDF 52 kb) [file 12864_2018_4768_MOESM1_ESM.pdf]

Table S1. Overrepresented biological process Gene Ontology terms from each developmental stage in each species.

Species	Stage	Gene Ontology Term	GO ID	p-value
C. rosaceus	Egg	cell communication	GO:0007154	5.74E-03
		cellular protein modification process	GO:0006464	4.85E-05
		cellular response to stimulus	GO:0051716	4.63E-03
		macromolecule modification	GO:0043412	4.85E-05
		protein modification process	GO:0036211	4.85E-05
		regulation of biological process	GO:0050789	4.63E-03
		regulation of cellular process	GO:0050794	4.63E-03
		signal transduction	GO:0007165	4.63E-03
		signaling	GO:0023052	5.74E-03
		single organism signaling	GO:0044700	5.74E-03
		single-organism cellular process	GO:0044763	6.55E-03
		single-organism process	GO:0044699	9.27E-03
C. subdepressus	Egg	biological regulation	GO:0065007	1.09E-04
		cell communication	GO:0007154	6.48E-08
		cell cycle process	GO:0022402	1.91E-03
		cellular macromolecule metabolic process	GO:0044260	3.34E-03
		cellular process	GO:0009987	5.56E-06
		cellular protein metabolic process	GO:0044267	1.76E-03
		cellular protein modification process	GO:0006464	4.94E-12
		cellular response to stimulus	GO:0051716	1.89E-07
		chromosome segregation	GO:0007059	3.83E-05
		macromolecule metabolic process	GO:0043170	3.73E-03
		macromolecule modification	GO:0043412	4.94E-12
		mitotic cell cycle process	GO:1903047	1.91E-03
		mitotic nuclear division	GO:0007067	1.91E-03
		nuclear division	GO:0000280	1.91E-03
		organelle fission	GO:0048285	1.91E-03
		organelle organization	GO:0006996	6.09E-03
		protein metabolic process	GO:0019538	2.08E-03
		protein modification process	GO:0036211	4.94E-12
		regulation of biological process	GO:0050789	1.89E-07
		regulation of cellular process	GO:0050794	1.89E-07
		response to stimulus	GO:0050896	6.92E-06
		signal transduction	GO:0007165	1.89E-07
		signaling	GO:0023052	6.48E-08
		single organism signaling	GO:0044700	6.48E-08
		single-organism cellular process	GO:0044763	7.40E-05

Table S1. Overrepresented biological process Gene Ontology terms from each developmental stage in each species.

Species	Stage	Gene Ontology Term	GO ID	p-value
C. rosaceus	Gastrula	amide biosynthetic process	GO:0043604	3.77E-36
		biosynthetic process	GO:0009058	6.01E-07
		cellular amide metabolic process	GO:0043603	3.77E-36
		aromatic compound metabolic process	GO:0006725	1.03E-03
		cellular biosynthetic process	GO:0044249	3.77E-36
		cellular component biogenesis	GO:0044085	1.44E-03
		cellular component organization or biogenesis	GO:0071840	2.86E-04
		cellular macromolecule biosynthetic process	GO:0034645	3.77E-36
		cellular macromolecule metabolic process	GO:0044260	2.15E-03
		cellular metabolic process	GO:0044237	3.54E-08
		cellular nitrogen compound biosynthetic process	GO:0044271	3.77E-36
		cellular nitrogen compound metabolic process	GO:0034641	1.08E-10
		cellular process	GO:0009987	1.46E-05
		chromosome organization	GO:0051276	1.32E-03
		gene expression	GO:0010467	2.17E-27
		heterocycle metabolic process	GO:0046483	1.03E-03
		macromolecule biosynthetic process	GO:0009059	3.77E-36
		macromolecule metabolic process	GO:0043170	4.19E-03
		metabolic process	GO:0008152	2.11E-05
		mRNA metabolic process	GO:0016071	6.20E-05
		mRNA processing	GO:0006397	6.20E-05
		neurological system process	GO:0050877	4.52E-03
		nitrogen compound metabolic process	GO:0006807	1.79E-10
		nucleic acid metabolic process	GO:0090304	3.59E-04
		nucleobase-containing compound metabolic process	GO:0006139	1.03E-03
		organic cyclic compound metabolic process	GO:1901360	1.03E-03
		organic substance biosynthetic process	GO:1901576	3.77E-36
		organonitrogen compound biosynthetic process	GO:1901566	3.77E-36
		organonitrogen compound metabolic process	GO:1901564	1.90E-28
		peptide biosynthetic process	GO:0043043	3.77E-36
		peptide metabolic process	GO:0006518	3.77E-36
		protein folding	GO:0006457	1.15E-07
		ribonucleoprotein complex biogenesis	GO:0022613	1.21E-10
		ribosome biogenesis	GO:0042254	4.15E-09
		RNA metabolic process	GO:0016070	1.04E-05
		RNA processing	GO:0006396	6.20E-05
		translation	GO:0006412	3.77E-36
C. subdepressus	Gastrula	amide biosynthetic process	GO:0043604	4.70E-17
		biosynthetic process	GO:0009058	1.99E-06
		catabolic process	GO:0009056	9.97E-03
		cellular amide metabolic process	GO:0043603	4.70E-17
		cellular aromatic compound metabolic process	GO:0006725	6.17E-03
		cellular biosynthetic process	GO:0044249	4.70E-17
		cellular component biogenesis	GO:0044085	9.24E-03
		cellular component organization or biogenesis	GO:0071840	4.99E-04
		cellular localization	GO:0051641	1.93E-03
		cellular macromolecule biosynthetic process	GO:0034645	4.70E-17
		cellular macromolecule localization	GO:0070727	1.94E-03
		cellular macromolecule metabolic process	GO:0044260	4.40E-03
		cellular metabolic process	GO:0044237	1.16E-07
		cellular nitrogen compound biosynthetic process	GO:0044271	4.70E-17

Table S1. Overrepresented biological process Gene Ontology terms from each developmental stage in each species.

Species	Stage	Gene Ontology Term	GO ID	p-value
C. subdepressus	Gastrula	cellular nitrogen compound metabolic process	GO:0034641	1.13E-08
		cellular protein localization	GO:0034613	1.94E-03
		chromosome organization	GO:0051276	1.15E-04
		endomembrane system organization	GO:0010256	3.36E-03
		establishment of localization in cell	GO:0051649	1.93E-03
		establishment of protein localization	GO:0045184	1.94E-03
		gene expression	GO:0010467	1.37E-16
		heterocycle metabolic process	GO:0046483	6.17E-03
		intracellular protein transport	GO:0006886	1.94E-03
		intracellular transport	GO:0046907	1.93E-03
		macromolecule biosynthetic process	GO:0009059	4.70E-17
		macromolecule localization	GO:0033036	1.94E-03
		macromolecule metabolic process	GO:0043170	3.15E-03
		metabolic process	GO:0008152	1.79E-06
		mRNA metabolic process	GO:0016071	5.10E-03
		mRNA processing	GO:0006397	5.10E-03
		ncRNA metabolic process	GO:0034660	3.61E-05
		nitrogen compound metabolic process	GO:0006807	9.31E-09
		nucleic acid metabolic process	GO:0090304	3.52E-03
		nucleobase-containing compound metabolic process	GO:0006139	6.17E-03
		organic cyclic compound metabolic process	GO:1901360	6.17E-03
		organic substance biosynthetic process	GO:1901576	4.70E-17
		organic substance transport	GO:0071702	1.94E-03
		organonitrogen compound biosynthetic process	GO:1901566	4.70E-17
		organonitrogen compound metabolic process	GO:1901564	1.54E-14
		peptide biosynthetic process	GO:0043043	4.70E-17
		peptide metabolic process	GO:0006518	4.70E-17
		plasma membrane organization	GO:0007009	3.36E-03
		protein folding	GO:0006457	8.62E-11
		protein localization	GO:0008104	1.94E-03
		protein targeting	GO:0006605	1.94E-03
		protein transport	GO:0015031	1.94E-03
		ribonucleoprotein complex biogenesis	GO:0022613	4.72E-10
		ribosome biogenesis	GO:0042254	3.56E-12
		RNA metabolic process	GO:0016070	6.93E-07
		RNA processing	GO:0006396	5.10E-03
		single-organism membrane organization	GO:0044802	3.36E-03
		single-organism metabolic process	GO:0044710	6.59E-03
		small molecule metabolic process	GO:0044281	3.25E-03
		translation	GO:0006412	4.70E-17
		tRNA metabolic process	GO:0006399	3.61E-05

Table S1. Overrepresented biological process Gene Ontology terms from each developmental stage in each species.

Species	Stage	Gene Ontology Term	GO ID	p-value
C. rosaceus	4-arm	amide biosynthetic process	GO:0043604	6.48E-04
		carbohydrate metabolic process	GO:0005975	5.54E-05
		cellular amide metabolic process	GO:0043603	6.48E-04
		cellular biosynthetic process	GO:0044249	6.48E-04
		cellular macromolecule biosynthetic process	GO:0034645	6.48E-04
		cellular nitrogen compound biosynthetic process	GO:0044271	6.48E-04
		macromolecule biosynthetic process	GO:0009059	6.48E-04
		neurological system process	GO:0050877	1.59E-03
		organic substance biosynthetic process	GO:1901576	6.48E-04
		organonitrogen compound biosynthetic process	GO:1901566	6.48E-04
		organonitrogen compound metabolic process	GO:1901564	7.99E-03
		peptide biosynthetic process	GO:0043043	6.48E-04
		peptide metabolic process	GO:0006518	6.48E-04
		system process	GO:0003008	9.03E-03
		translation	GO:0006412	6.48E-04
		transmembrane transport	GO:0055085	1.96E-04
C. subdepressus	4-arm	biological regulation	GO:0065007	6.63E-03
		cell communication	GO:0007154	2.90E-03
		cell cycle	GO:0007049	8.55E-13
		cell cycle process	GO:0022402	1.02E-06
		cell division	GO:0051301	7.26E-03
		cellular aromatic compound metabolic process	GO:0006725	5.13E-03
		cellular component organization	GO:0016043	4.04E-04
		cellular component organization or biogenesis	GO:0071840	9.96E-04
		chromosome organization	GO:0051276	3.93E-06
		chromosome segregation	GO:0007059	1.26E-05
		DNA metabolic process	GO:0006259	1.06E-05
		heterocycle metabolic process	GO:0046483	5.13E-03
		mitotic cell cycle	GO:0000278	1.21E-09
		mitotic cell cycle process	GO:1903047	1.02E-06
		mitotic nuclear division	GO:0007067	1.02E-06
		nuclear division	GO:0000280	1.02E-06
		nucleic acid metabolic process	GO:0090304	2.70E-03
		nucleobase-containing compound metabolic process	GO:0006139	5.13E-03
		organelle fission	GO:0048285	1.02E-06
		organelle organization	GO:0006996	1.19E-07
		organic cyclic compound metabolic process	GO:1901360	5.13E-03
		signaling	GO:0023052	2.90E-03
		single organism signaling	GO:0044700	2.90E-03
		transmembrane transport	GO:0055085	5.75E-05
C. rosaceus	8-arm	NA	NA	NA
C. subdepressus	8-arm	cellular metabolic process	GO:0044237	3.65E-03
		lipid metabolic process	GO:0006629	9.18E-03
		metabolic process	GO:0008152	6.99E-03
